# Development of Nanoemulsion Based Gel Loaded with Phytoconstituents for the Treatment of Urinary Tract Infection and *in Vivo* Biodistribution Studies

**DOI:** 10.15171/apb.2017.073

**Published:** 2017-12-31

**Authors:** Atinderpal Kaur, Sonal Gupta, Amit Tyagi, Rakesh Kumar Sharma, Javed Ali, Reema Gabrani, Shweta Dang

**Affiliations:** ^1^Department of Biotechnology, Jaypee Institute of Information Tehnology, A-10, Sector 62, Noida, UP 201307, India.; ^2^Department of Nuclear Medicine, Institute of Nuclear Medicine and Allied Sciences, Brig SK Mazumdar Marg, Delhi, 110054, India.; ^3^Division of CBRN Defence, Institute of Nuclear Medicine and Allied Sciences, Brig SK Mazumdar Marg, Delhi, 110054, India.; ^4^Faculty of Pharmacy, Jamia Hamdard, Hamdard Nagar, New Delhi, 110062, India.

**Keywords:** Cranberry, Gamma scintigraphy, Intravaginal drug delivery, Polyphenon 60, Radiolabelling, Urinary tract infection

## Abstract

***Purpose:*** A nanoemulsion based gel containing Polyphenon 60 (P60) and cranberry (CRB) has been developed to deliver via intravaginal route for the treatment of urinary tract infection.

***Methods:*** Polyphenon 60 and cranberry were loaded in a single nanoemulsion gel (NBG) by ultra-sonication method and characterized for particle size, rheological properties, in vitro release and growth curve analysis. P60+CRB NBG were radiolabelled using technetium pertechnetate (^99m^Tc) to perform in vivo pharmacokinetic studies in animals.

***Results:*** The finalized NE had a droplet size of 58±1 nm. In vitro release of 90.92 ± 0.6% in 8 hr for P60 and 99.39 ± 0.5% in 6 hr for CRB was observed in simulated vaginal fluid. Growth curve of E. coli indicated the inhibitory action of nanoemulsion based gel at the fifth hour of inoculation. Gamma scintigraphy studies on female Sprague-Dawley rats showed transport of nanoemulsion based gel from the vaginal cavity into the systemic circulation. Further, biodistribution studies with radiolabelled P60+CRB NBG showed significant higher uptake of radiolabelled actives by kidney (3.20±0.16) and urinary bladder (3.64±0.29), when administered intravaginally.

***Conclusion:*** The findings suggested ^99m^Tc-P60+CRB NBG can potentially be transported through vaginal cavity and reach the target organs and showed effective distribution in organs affected in urinary tract infection

## Introduction

Urinary tract infection (UTI) is the most common type of infection occurring amongst women caused by *E. coli*. Antibiotic therapy is used as a standard treatment for UTI, however rising rates of reoccurrence and resistance to antibiotics has led to consideration of natural plant products as alternative treatment.^1^ Cranberry (CRB), *Vaccinium macrocarpon,* is well known for its use in urinary tract infection (UTI) for many years.^2^ CRB is particularly known to exert anti-adhesive effect against P-fimbriated bacteria by releasing certain adhesion factors that do not allow bacteria to adhere on the surface.^3^ Green tea catechins (GTCs) are also known to exhibit a range of pharmacological and biological effects like anti-microbial,^4^ anti-inflammatory and many more.^5,6^ Most of the polyphenols present in green tea are flavanols that can be categorised as catechins^7^ and are accountable for anti-microbial activity.^4^ GTCs are reported to exert antibacterial action by binding to the outer cell membrane and cause cell leakage leading to ultimate cell lysis/death.^8^ However, gram negative bacteria are less susceptible to GTCs due to the presence of an additional lipopolysaccharide layer over their cell membrane.^9^

The combination therapy has been investigated to prevent emergence of resistant strains and to lower down the concentration of individual agents thus minimizing the likelihood of dose-related toxicity.^10^ The therapeutic efficacy of natural compounds can be improved by their incorporation in suitable delivery systems. With advances in the field of nano medicine, nanotechnology based formulations are being explored for intravaginal delivery. Nanoemulsions (NEs) have shown to enhance the solubility, evade the enzymatic attack and thus increase the bioavailability and prolong the shelf life by protection against oxidation and hydrolysis.^11^ NEs serve as a versatile carrier for drug delivery owing to their lipophillic, hydrophilic and amphiphillic phases.^12^ Vaginal formulations for local delivery have been reported.^13^ However, the aspect of systemic delivery via vaginal route has not been reported widely; fate and stability of active agents crossing the vaginal mucosa being a major concern. Prolonged residence time of vaginal formulation inside the cavity is desired because of leakage and redistribution of vaginal fluids within the cavity.^13^ For systemic delivery, it is of prime importance that formulations either adhere for considerable time or cross the vaginal lining as soon as possible as it is instilled in the vaginal cavity. To overcome these limitations, a chitosan based muco-adhesive polymeric gel of NE was prepared hypothesizing increased drug contact time with infected tissue and improved therapy.^14^ Chitosan is a bio-polymer that exhibits good biodegradability, low toxicity and bio-adhesiveness which makes it an appropriate agent to be used in drug delivery systems.^15^

The aim of the present work was to prepare nanoemulsion based gel formulation for GTCs and cranberry powder for intravaginal delivery. A comparative oral vs intravaginal study was planned to study the path and biodistribution of P60+CRB NBG by radiolabelling it with ^99m^Tc on female Sprague-Dawley rats.

## Materials and Methods

Polyphenon 60 or green tea catechins (GTCs) and reagent grade Tween 20 was obtained from Sigma Aldrich (Bangalore, India). Cranberry powder was generously gifted by Naturex, DBS, New Jersey (U.S.A). Oleic acid and glycerol was a product of CDH (P) Ltd, India. Water used was Milli-Q (Millipore, USA).

### Animal Preparation

Female Sprague Dawley rats (aged 3-4 months) weighing 180-200 g was obtained from the Central Animal House Facility of INMAS, Delhi, India. Rats were kept at normal room temperature of 25 ± 5°C.

### Formulation of Polyphenon 60 and Cranberry nanoemulsion

The solubility of polyphenon 60 and cranberry were checked in different oils, surfactants and co-surfactants. The nanoemulsion was prepared by dissolving cranberry in oleic acid as oil phase, tween 20 as surfactant and glycerol as a co-surfactant. P60 was dissolved in milli-Q water to prepare aqueous phase and added drop wise to the oil phase with continuous stirring to form pre emulsion. This pre-emulsion was subjected to high shear homogenization using Tissue Master 125 homogenizer (Omni International, Georgia) at 10,000 rpm for 20 min under ice bath and further subjected to high energy ultra-sonication via Bench Top Ultrasonicator (Model UP400S, 24 KHz 400 W, Hielcher, Ultrasound Technology, Germany) at amplitude of 70% for 286 sec at 0.3s ON and 0.7s OFF cycles. The prepared nanoemulsion was characterized for particle size and zeta potential determination using Malvern Zetasizer (Malvern, Worcestershire, UK). Before determining particle size and zeta potential, the nanoemulsion was diluted with HPLC water at ratio of 1:50 v/v.

### Development and characterization of Nanoemulsion based gel

#### Preparation of Nanoemulsion based Gel 

Different concentrations of Chitosan (1%) were mixed in lactic acid (1%) because it provides simulated vaginal medium pH for chitosan to dissolve easily at vaginal pH,^15^ and kept overnight to ensure complete hydration and was then added to NE formulation with continuous mixing using a magnetic stirrer until a homogeneous dispersion was achieved.

#### Rheological characterization of gels

Chitosan gels were first evaluated in terms of pH and phase separation/turbidity to identify the most suitable gel for vaginal application. pH values were measured by pH meter (Thermo Orion 420A+ Basic pH meter) after dilution of the gel formulation in ultrapure water (1:10, w/v). Formulations were then subjected to rheological characterization by using Modular Compact Rheometer MCR302 (Anton Paar, Austria). The rheological measurements were performed at 37°C, after a 5 min rest time. Viscosity curves were obtained between 0.01 and 100 s^-1^ shear rates to study the flow behaviour of different gels. Storage modulus G′ (that describes elastic properties) and loss modulus G″ (that describes viscous properties) were recorded at frequencies ranging between 0.1 and 10 Hz. The loss tangent (tan δ) was calculated as the G″ to G′ ratio (measure of the ratio of energy lost to energy stored during gel deformation). All these parameters relate with the stability, spreading and retention properties of prepared gels.

#### Growth Curve Analysis

The effect of P60+CRB and their formulations on the growth of bacteria over a time period of 20 hr were studied by plotting the bacterial growth curve. To examine the growth curve of bacteria, bacterial cells adjusted to final concentration of 5×10^5^ cfu/ml, were exposed to different formulations including aqueous form of P60+CRB, their NE formulation, corresponding placebo (P_NE_), NE based gel of P60+CRB and corresponding placebo (P_NBG_), at their respective MIC values that were observed in our previous work^16^ as (3.3 mg/ml) for P60 and (10 mg/ml) for CRB, respectively. Each culture was incubated in a shaking incubator at 37°C and absorbance was measured at 595 nm at different time intervals (0, 5, 10, 15 and 20 hr) to obtain growth curve for the bacteria.^17^

#### In vitro drug release (Ex vivo release profile from porcine vaginal mucosa) 

Porcine vaginal mucosa was obtained from a slaughter house and approximately 2.5 cm^2^ area was sliced out with sharp blade, excess fat was trimmed and slices of about 450 mm thickness were prepared. These slices were hydrated in simulated vaginal fluid (SVF) (acetate buffer with pH 4.2: was prepared by dissolving sodium acetate 13.6 g and 6 ml until pH 4.2 of acetic acid in 1000 ml of distilled water)^14^ until used. 5 mg of gel was applied on vaginal mucosa (2 cm^2^ area) that is tied to the lower end of donor compartment. The volume of the receptor compartment was kept 10 ml. The cell was assembled in such a way that the mucosal surface was just flushed with the SVF (pH 4.2) maintained at 37°C and stirred continuously on a magnetic stirrer at 50 rpm. Aliquots of 1 ml were withdrawn at pre-determined time intervals and analyzed for P60 and CRB content after suitable dilutions by UV spectrophotometric method. The volume of fluid was replaced with the same volume of simulated vaginal fluid after each sampling to maintain the sink conditions. The percentage of drug permeated across the vaginal mucosa was calculated as in equation 1.

%Release= {(conc. of drug × Vol. of dissolution media × dilution factor) / Dose} × 100 ** (**Equation 1)

### In vivo Pharmacokinetic studies

#### Radiolabeling of aqueous form of P60 and CRB and its nanoemulsion based gel

P60+CRB NBG were radiolabeled by the incorporation of short half-life gamma emitting radionuclide like Technetium-99m (^99m^Tc). Aqueous form of P60+CRB and its NE based gel were radiolabeled with ^99m^Tc using direct labelling method.^18^ 500 μl of aqueous form of P60 (3 mg) in acetone was taken and mixed with 200 μl of stannous chloride dihydrate solution (2 mg/ml in ethanol). To the resultant mixture (filtered through 0.22 micron nylon filter) 500 μl of ^99m^Tc (5 mCi) was added with continuous mixing and incubated at 37°C for 30 min. The resultant formulations obtained had 100 μCi/20 μl activities. The radiolabelled P60 was then mixed with CRB NE based gel. The radiolabeling efficiency (%) was determined by using instant thin layer chromatography-silica gel strips (ITLC-SG, Gelman Sciences, Inc., Ann Arbor, MI USA) using acetone as mobile phase. *In vitro* stability of radiolabeled formulation was evaluated and optimized in normal saline as well as in blood plasma.^19^ The effect of concentration of stannous chloride and incubation time on radiolabeling efficiency was studied to achieve optimum reaction conditions by using the equation 2.

% Radiolabelling= (Radioactive counts retained in lower half of strip / Total radioactive counts retained in the strip) ×100 (Equation 2)

#### Gamma scintigraphy imaging 

The Sprague-Dawley rats (female, aged 2-3 months) were selected for the study. The rats were anesthetized using 0.4 ml ketamine hydrochloride intraperitonial injection (50 mg/ml). The rats were divided into three groups (3 rats in each group). (n=9)

Group I: Rats were administered with ^99m^Tc-P60+CRB NBG (orally);

Group II: Rats were administered with ^99m^Tc-P60+CRB NBG (intra vaginally) and

Group III: Rats were administered with ^99m^Tc-P60+CRB Aqueous form (intra vaginally).

Each rat was given 0.6 μl radiolabelled NBG containing concentration of (100 μCi/20 μl) equivalent to (3 mg of P60 and 1 mg of CRB in 0.6 μl, respectively) with the help of catheter made up of low density polyethylene tubing (LDEP) of internal diameter 0.1 mm. Anesthetized rats were then placed on the imaging platform and imaging was performed using Single photon emission computerized tomography (SPECT, LC 75-005, Diacam, Siemens AG; Erlanger, Germany) gamma camera.

#### Biodistribution Studies 

The rats were divided into three groups as following: 3 rats per time point per group (n=36)

Group I: Rats administered with ^99m^Tc-P60+CRB NBG orally

Group II: Rats administered with ^99m^Tc-P60+CRB NBG intravaginally

Group III: Rats administered with ^99m^Tc-P60+CRB aqueous intravaginally

Each group contains 12 rats. 3 rats were sacrificed at each time interval of the study. Prior to the administration of formulations, the rats were anesthetized using 0.4 ml ketamine hydrochloride intraperitonial injection (50 mg/ml). Blood samples were collected by retro-orbital vein puncture from each rat at predetermined time points (0.5, 3, 6 and 24 h) post-administration of formulations. The rats were sacrificed by cervical dislocation at different time intervals. Subsequently, urinary tract organs (kidney, spleen and urinary bladder along with ureters) were dissected, washed twice using normal saline, made free from adhering tissue/fluid, and weighed. Radioactivity present in each tissue/organ was measured using shielded well-type gamma scintillation counter. Radio pharmaceutical uptake per gram in each tissue/organ was calculated as a fraction of administered dose using equation 3.

Radioactivity %/g of tissue **=** (counts in sample × 100) / (wt. of sample × Total counts injected) (Equation 3)

Pharmacokinetic parameters for P60+CRB NBG formulation were calculated.^20^ Kidney and Urinary bladder were selected as target organs and their organ targeting efficiency was calculated using two equations (5 and 6) mentioned below.^21^ Drug targeting efficiency (DTE %) represents time average partitioning ratio was calculated as follows:

DTE %={( AUC target organ/ AUC blood) Ivag / (AUC target organ/ AUC blood) oral} × 100 (Equation 4)

Direct transport percentage (DTP %) of target organ was calculated using equation,

DTP %={(Bivag- Bx) / Bivag} × 100 (Equation 5)

Where B_x_= (B_oral_/P_oral_) × P_ivag_. *B*_x_ is the target organ AUC fraction contributed by systemic circulation following oral administration,

*B*oral is the AUC_0–24h_ (target organ) following oral administration,

*P*_oral_ is the AUC_0–24h_ (blood) following oral administration,

*B*_ivag_ is the AUC_0–24h_ (target organ) following intravaginal administration,

*P*_ivag_ is the AUC_0–24h_ (blood) following intravaginal administration,

AUC is the area under the curve.

#### Data Analysis

Results of *in vitro* drug release and biodistribution data were reported as mean± SD (n=3), and the difference between the groups were tested using two-way ANOVA using Graph Pad Prism 5.0 and data analysis tool in Microsoft Excel.

## Results

### Preparation of P60+CRB Nanoemulsion 

Optimized NE was formulated with combined level of drug content 41 mg/ml (P60=11 mg/ml; CRB=30 mg/ml), oil content 5% w/w, emulsifier content 16.4% w/w (Table 1). The time of sonication and amplitude was optimised to be 300 s and 30% respectively. Particle size analysis showed particles with 58 nm size, PDI of 0.2 and zeta potential of -16 mV.

**Table 1:** Conditions and quantities of drugs and excipients selected for formulation of Nanoemulsion and characterization

**Nanoemulsion**		
**Drug candidate**	**P60+CRB**	**Composition**
**Oil**	Oleic Acid	10%
**Surfactant**	Tween 20	20%
**Co-surfactant**	Glycerol	3.52%
**Aqueous Phase**	Mili Q water	66.48%
**Label claim**	41 mg/ml	P60 = 11 mg/ml
		CRB = 30 mg/ml
**Formulation Parameters**		
**Homogenization Speed**		10,000 rpm
**Homogenization Time**		30 min
**Time of Ultrasonication**		300 sec
**% Amplitude**		30%
**Charaterization Parameters**		
**Droplet size**		58±1 nm
**PDI**		0.2±0.015
**Zeta potential**		-16±0.2 mV

Where, P60: Polyphenon 60; CRB: Cranberry; PDI: Particle distribution index

### Development and characterization of Nanoemulsion based gel

Chitosan at three different concentrations was used to prepare the nanoemulsion based Gels (NBG). Chitosan gel (1%), hydrated in lactic acid (1%) was selected primarily based on their clarity and pH value that is close to the physiological (vaginal pH=3.5-4.5) conditions. (Table 2) represented the final composition and pH values of all the prepared chitosan gels.

**Table 2:** Composition of different gels with their pH values and homogeneity

**Ingredients** **(for 10g of gel)**	**Formulation codes**		
	**CH 1%**	**CH 1.5%**	**CH 2%**
Chitosan (g)	0.10	0.15	0.20
Lactic acid (ml)	1.15	0.15	0.20
Nanoemulsion (ml)	8.85	8.85	8.85
pH	3.2±0.2	3.7±0.2	4.9±0.1
Homogeneity	**✓**	**✓**	**✓**

Where, CH is the different concentration of Chitosan containing formulations

#### Rheological studies of selected nanoemulsion based gel

**Figure 1:**
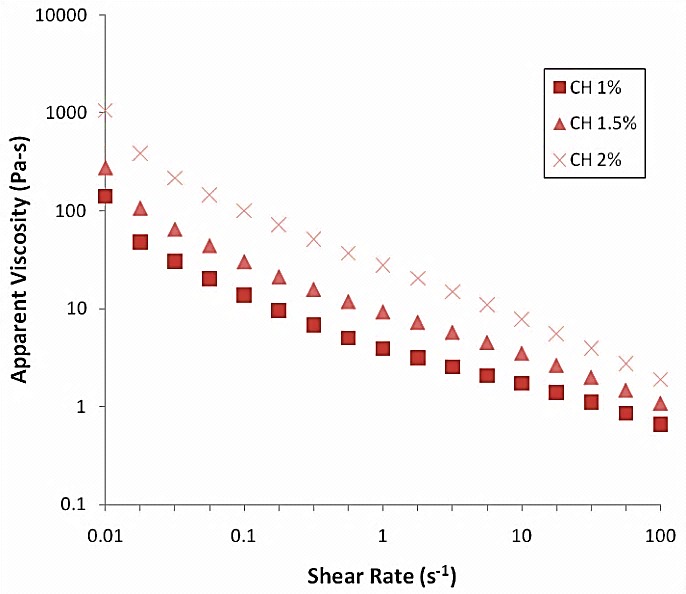
Viscosity profiles as a function of shear rate for tested gels prepared with different concentrations of chitosan CH 1%, CH 1.5%, CH 2%.

**Figure 2:**
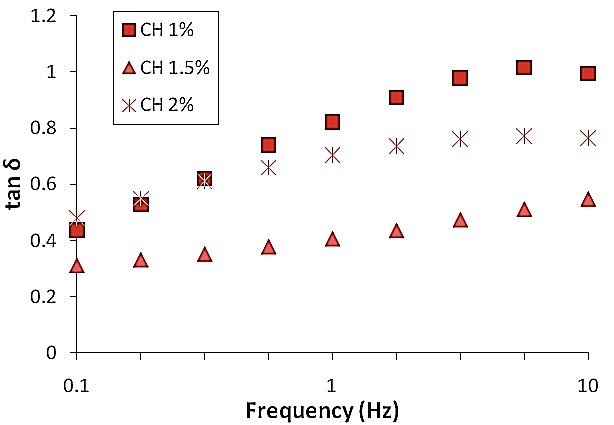
tan δ as a function of frequency for tested gels prepared with different concentrations of chitosan CH 1%, CH 1.5%, CH 2%.

The apparent viscosity profiles of all the selected gels are presented in (Figure 1). All three gels presented a non-Newtonian, pseudo-plastic behaviour, which is a characteristic of polymeric systems. Results showed viscosity values from around 141 Pa.s up to 1060 Pa.s at 0.01 s^-1^, decreasing down to approximately 0.5-2 Pa.s at 100 s^-1^. (Figure 2) presents its variability for all the three gels along the considered frequency range (0.1 to 10 Hz). Higher elastic component (lower values of tan δ) observed for CH 1.5% gel, could favour its ability to stay in place after being administered.

#### In vitro analysis of nanoemulsion based gel

**Figure 3:**
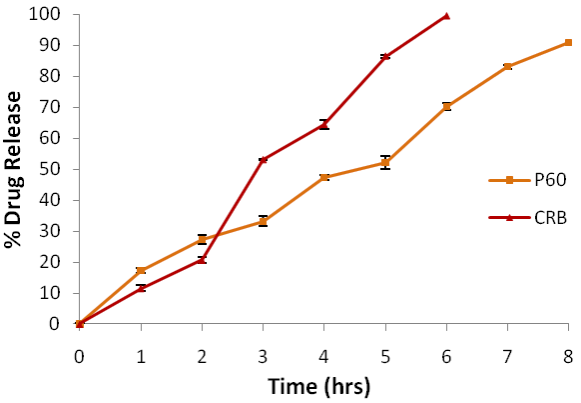
Percentage release of Polyphenon 60 and Cranberry from Nanoemulsion based gel across Porcine Vaginal Mucosa via Franz Diffusion Cell

Drug Release Studies: The *ex- vivo* release profile of prepared nanoemulsion gel was performed in simulated vaginal media using porcine vaginal mucosa. (Figure 3) showed maximum release of 90.92 ± 0.6% in 8 hr for P60, while CRB showed 99.39 ± 0.5% release within 6 hr. This sustained release pattern for both the P60 and CRB from NBG indicated that active was released from the polymeric matrix and gel in the simulated vaginal fluid.

**Figure 4:**
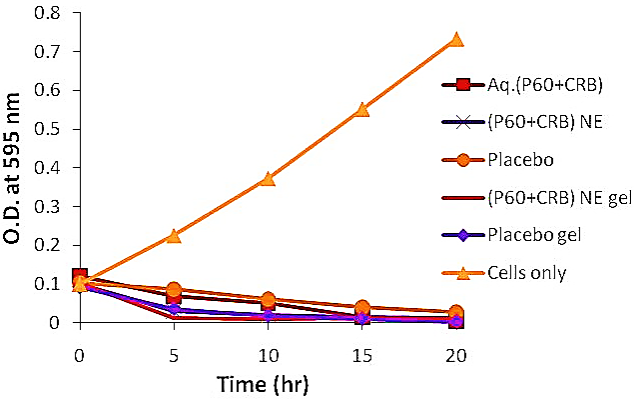
Growth Curve of *E. coli* when exposed to different formulations at 37°C over a time period of 0 hr-20 hr

Antibacterial potential of nanoemulsion based gel with respect to time: The graph (Figure 4) indicates that the aqueous P60+CRB could inhibit the growth of *E. coli* at 15 hr while the NE formulation of P60+CRB and its NBG showed the sudden drop in the turbidity of bacterial culture at the 5^th^ hr of inoculation.

#### In vivo pharmacokinetic analysis of nanoemulsion based gel

P60 was radiolabeled using ^99m^Tc and optimum SnCl_2_.2H_2_O concentration was found to be 2 mg/ml with an incubation time of 30 min. Radiolabelled P60 was mixed with cranberry NBG to prepare radiolabelled P60+CRB NBG. Maximum labelling efficiency of aq. P60+CRB and P60+CRB-NBG was found to be 95.32 ± 0.15 % and 92.62 ± 0.09 %, respectively. Further the *in vitro* stability of radiolabelled formulation was checked in normal saline and blood serum which was found to be 90.25 ± 0.10 % and 94.64 ± 0.21 % respectively.

#### Gamma scintigraphy imaging 

**Figure 5:**
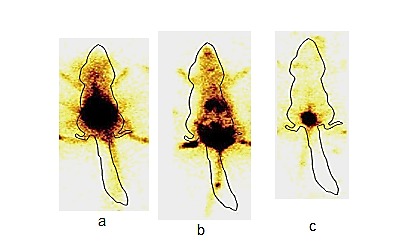
Gamma scintigraphy images of (a) oral ^99m^Tc-P60+CRB NBG, (b) intra-vaginal ^99m^Tc-P60+CRB NBG, (c) intra-vaginal ^99m^Tc-Aq. P60+CRB showing presence of radioactivity in different organs

Among the three groups scintigraphy images as shown in (Figure 5b) post intra-vaginal administered ^99m^Tc-P60+CRB-NBGrats indicated the rapid distribution and maximum systemic bioavailability of radiolabelled formulation within urinary tract as complete coverage of vaginal mucosa and urinary bladder was seen. While scintigraphy images of post intra-vaginal administered ^99m^Tc-P60+CRB rats as shown in (Figure 5c) indicated local distribution and minimal systemic distribution of the formulation. However the scintigraphy images (Figure 5a) of orally administered ^99m^Tc-P60+CRB-NBGrat showed maximum distribution in GIT as compared to the urinary tract.

#### Biodistribution Analysis

In this analysis distribution of radiolabelled drug in target organs after administration of ^99m^Tc-P60+CRB NBG and ^99m^Tc-P60+CRB aqueous form following vaginal and oral administration in Sprague-Dawley rats were compared. The radioactivity in percentage per gram of the total administered dose was estimated at pre-determined time intervals up to 24 hr (Table 3). The concentration of radiolabelled drug in blood at different sampling time points for different formulations was calculated. The analysis indicated that ^99m^Tc-P60+CRB NBG given intravaginally and orally reached the systemic circulation in 3 hr as compared to the aqueous formulation given vaginally. The percent per gram concentration of ^99m^Tc-P60+CRB NBG in kidney and urinary bladder were found to be (3.20±0.16) and (3.64±0.29), respectively following the vaginal administration, which was significantly higher as compared to both ^99m^Tc-P60+CRB NBG administered orally (1.82±0.32) for kidney and (0.91±0.21) for urinary bladder and ^99m^Tc-P60+CRB aqueous form administered vaginally (1.21±0.28) for kidney and (1.88±0.14) for urinary bladder. Whereas no comparative difference at different time points in concentration of drug in the spleen was observed after administration of radiolabelled drug. The ^99m^Tc-P60+CRB NBG maintained its concentration systemically up to 24 hr when given vaginally. Pharmacokinetic parameters for the ^99m^Tc-P60+CRB NBG and ^99m^Tc-P60+CRB aqueous form were also calculated (Table 4). The target organs showed the *C*max_kidney_ (3.20 %/g) and Cmax_urinary bladder_ (3.64 %/g) at 3 hr following vaginal administration as compared to oral administration showed *C*max_kidney_ (2.01 %/g) and Cmax_urinary bladder_ (1.08 %/g) at 6 hr was achieved, which may also be attributed to maximum target drug delivery in minimum time following vaginal administration of ^99m^Tc-P60+CRB NBG. This confirmed that nano sized gel adhered to the mucosa and crossed epithelium to reach the target organs when given vaginally as compared to oral route. The Area Under Curve (AUC) of kidney and urinary bladder after administration of ^99m^Tc-P60+CRB NBG (intravaginal), was found to be (68.27 hr %/g) and (59.30 hr %/g), respectively, which was significantly higher as compared to AUC_kidney_ (33.22 hr %/g) and AUC_urinary bladder_ (56.95 hr %/g) calculated following administration of ^99m^Tc-P60+CRB NBG (orally) and AUC_kidney_ (20.95 hr %/g) and AUC_urinary bladder_ (23.5 hr %/g) after ^99m^Tc-P60+CRB aqueous form administered vaginally. DTP% and DTE% were also calculated which represent the percentage of drug directly transported to the target organs via the vaginal pathway using tissue/organ distribution data (Table 5). The results showed the DTE%_kidney_ (162.98), DTE%_urinary bladder_ (249.15) and DTP%_kidney_ (38.64), DTP%_urinary bladder_ (59.67) for target organs via vaginal administration of ^99m^Tc-P60+CRB NBG. The findings suggested that ^99m^Tc-P60+CRB NBG administered vaginally had better target organ efficiency.

**Table 3:** Distribution of 99mTc-P60+CRB NBG (orally and Ivag), 99mTc-P60+CRB aqueous form (Ivag) at Different Time Intervals in Sprague-Dawley female Rats.

**Formulation and route of drug administration**	**Distribution of radiolabelled P60+CRB in different organs at different sampling time points**				
	**ORGAN**	**0.5 hr**	**3 hr**	**6 hr**	**24 hr**
**Oral 99mTc-P60+CRB NBG**	BLOOD	0.46±0.12	1.49±0.22	2.06±0.17	0.38±0.19
	KIDNEY	0.73±0.21	1.82±0.3	2.01±0.15	0.67±0.19
	URINARY BLADDER	0.23±0.15	0.91±0.21	1.08±0.30	0.42±0.12
	SPLEEN	0.49±0.13	0.99±0.15	1.20±0.22	0.73±0.11
**Ivag99mTc-P60+CRB NBG**	BLOOD	0.65±0.20	1.69±0.14	2.12±0.25	0.90±0.23
	KIDNEY	1.44±0.25*	3.20±0.16*	3.09±0.35*	1.83±0.18*
	URINARY BLADDER	2.87±0.35*	3.64±0.29*	3.12±0.27*	1.36±0.19*
	SPLEEN	0.32±0.08	1.36±0.13	1.58±0.17	0.82±0.09
**Ivag99mTc P60+CRB Aqueous form**	BLOOD	0.31±0.10	0.97±0.20	0.84±0.13	0.33±0.10
	KIDNEY	0.46±0.11	1.21±0.28	1.10±0.20	0.60±0.10
	URINARY BLADDER	2.30±0.24	1.88±0.14	0.94±0.09	0.56±0.10
	SPLEEN	0.30±0.10	0.68±0.11	0.52±0.08	0.43±0.1

Each value is the mean ± SD of three estimations. Radioactivity was measured at 0.5 hr, 3 hr, 6 hr and 24 hr. Only statistically significant outcomes at p<0.05 have been reported with *. *The percentage per gram count of radioactivity in kidney and urinary bladder observed after intravaginal administration showed significant difference as compared to radioactivity observed after oral administration of gel and intravaginal administration of aqueous formulation.

**Table 4:** Pharmacokinetics of 99mTc-P60+CRB NBG (orally and Ivag) and 99mTc-CRB aqueous form (Ivag) at Different Time Intervals in Sprague Dawley Rats.

**Formulation and route of administration**	**ORGAN**	**Cmax (%/gm)**	**Tmax (hr)**	**AUC**_0-24 hr_
**Oral 99mTc-P60+CRB NBG**	BLOOD	2.26	6	31.925
	KIDNEY	2.01	6	33.22
	URINARY BLADDER	1.08	6	18.6
	SPLEEN	1.20	6	22.62
**Ivag99mTc-P60+CRB NBG**	BLOOD	2.52	6	40.26
	KIDNEY	3.20*	3	68.27*
	URINARY BLADDER	3.64*	3	59.30*
	SPLEEN	1.58	6	28.19
**Ivag99mTc P60+CRB Aqueous form**	BLOOD	0.97	3	14.92
	KIDNEY	1.21	3	20.955
	URINARY BLADDER	2.30	0.5	23.52
	SPLEEN	0.68	3	11.64

Only statistically significant outcomes at p<0.05 have been reported with *. *AUC:* area under the curve, *CRB:* Cranberry, *P60:* Polyphenon 60, *Ivag:* intravaginal, *NBG:* Nanoemulsion based gel.*The Cmax and AUC of radiolabelled NBG administered via intravaginal route in kidney and urinary bladder showed significant difference as compared to other groups of study.

**Table 5:** Drug targeting efficiency and direct target organ transport following intravaginal administration of 99Tc-P60+Cranberry Nanoemulsion based gel.

**Formulation and route of administration**	**Target organ**	**Drug target efficiency (DTE %)**	**Direct target organ transportation (DTP %)**
P60+CRB NBG Ivag	Kidney	162.98	38.64
P60+CRB NBG Ivag	Urinary bladder	249.15	59.67

Where *ivag*: intravaginal, *NBG*: Nanoemulsion Based Gel, *P60:* Polyphenon 60, *CRB:* Cranberry.

## Discussion

Ease of administration, bypass of hepatic metabolism and achieving systemic concentration in therapeutic ranges at lower doses make intravaginal delivery a lucrative route of administration. Cicinelli^22^ reported that the vagina has specific blood flow characteristics, either by venous and lymphatic channels or by portal type circulation that allows bypassing the GIT -absorption and liver detoxification and facilitate the transport of drug molecules from vagina to the uterus and systemic circulation.

Lai et al.^23^ reported that polymeric nanoparticles of particle size less than 500 nm can distributed in cervical-vaginal regions and rate of transport across the vaginal mucosa depend highly upon the surface properties of nano-carriers. Particles in micro meter range were reported to be too large to cross the mucosa.

Hypothesizing the effect of particle size, nanoemulsions of Green tea and cranberry were prepared in the present study and for enhanced residence time, a bio adhesive gel using chitosan was prepared. As reported by das Neves et al.^24^ most of the vaginal formulations were administered to animal models by making a dispersion of the same in PBS; however, aqueous based gel is a feasible and acceptable option for human administration. The gel not only provides a medium to carry the actives, it also provides a three dimensional matrix which can form interlocks with the mucin layers of the cervical–vaginal region. Lin et al.^25^ and Abbas et al.^26^, reported administration of thermo sensitive gels for PLGA nanoparticles and for delivery of plasmid DNA respectively. In our previous work, we have established synergy between green tea catechins and CRB.^16^ Catechins and Proanthocyanidins are two classes of secondary metabolites that are characterized by a common O-heterocyclic structure and are reported to be used as anti-bacterial agents.^27^ To prepare CRB and P60 loaded NE system, a set of variables was generated that could result in minimum particle size, PDI and zeta potential. Besides, it would be desirable to encapsulate highest possible content of P60 and CRB with minimum amount of emulsifier. Upon formulating it was observed that both the criteria did not lead to expected particle size range. Therefore, higher content of emulsifier was used so as to solubilise high content of P60 and CRB and got adequately adsorbed on the interface. Similarly, while deciding between % amplitude and time of sonication, preference was given to % amplitude to be kept as minimum to avoid degradation of actives. The optimized NE (closest to the predicted values) was achieved by combining the 41mg/ml (P60=11 mg/ml; CRB=30 mg/ml) drug content, 5% w/w oil content, 16.4% w/w emulsifier content, 31% of amplitude and 286 s time of sonication. . The values of MDS, PDI and zeta potential were found to be in good agreement with the previous findings from literature. Hielscher^28^ reported the use of minimum % amplitude while studying the effect of ultrasonication process parameters.

With the aim to enhance the residence time chitosan gels were prepared. Rheological characterization of all three gels presented a non-Newtonian, pseudo-plastic behaviour.^23^ Newtonian flow was not observed at varying shear stress values. 1.5% chitosan gel showed higher elastic component (lower values of tan δ).

The sharp drop in turbidity of bacterial culture as exhibited by NBG of P60+CRB could be attributed to nano-sized droplets besides, NH^3+^ groups on protonated chitosan interact electrostatically with negatively charged phospholipids present in cellular membranes of bacteria resulting in leakage of intracellular material. Costa et al.^29^ also studied the effect of chitosan at low pH on *E. coli* and showed an increase in inner and outer permeability of *E. coli* cellular membrane. *Ex vivo* release model comprising porcine vaginal mucosa showed maximum release of 90.92 ± 0.6% in 8 hr for P60 and 99.39 ± 0.5% release for CRB within 6 hr implying complete release of actives in simulated vaginal media.

To investigate the transport of the optimized ^99m^Tc-P60+CRB-NBG via vaginal route, gamma scintigraphy and biodistribution studies were conducted. Gamma scintigraphy images showed significant distribution of radiolabelled NBG formulation administered intravaginally as compared to formulation administered orally and aqueous drug administered intravaginally. In a similar study by Mehta et al.^30^ pellets (filled into hard gelatin capsule) and cetomacrogol cream, both labeled with Indium-111 DTPA (for gamma scintigraphy) were evaluated for intravaginal distribution and retention over a 24 hr period. From the results it was found that there was complete distribution of creams after 1 hr of administration in the vaginal system and complete coverage of vaginal mucosa was observed. The radiolabelled drug also retained in the vaginal mucosa slightly at 24 hr. Our obtained scintigrams also showed that radiolabelled gel retained for 24 hr after intravaginal administration. A higher uptake of percentage per gram of ^99m^Tc-P60+CRB-NBG into systemic circulation and target organs, kidney and urinary bladder was observed as compared to ^99m^Tc- P60+CRB aqueous form after vaginal administration. The DTP% and DTE% was also observed higher, which could be due to mucoadhesive nano emulsion based gel formulation. It is evident from biodistribution results that the ^99m^Tc-P60+CRB-NBG could cross the vaginal mucosa substantially to reach the organs infected by *E. coli* in Urinary Tract infections. In a similar *in vivo* study performed by Ilem-Ozdemir et al.^31^ alendronate was labelled with ^99m^Tc by direct method and a comparative study was done for intravaginal and intravenous routes. Results suggested that the labeled alendronate could cross the vaginal mucosa and there was an uptake of drug by bone tissues upon intra-vaginal administration. Hanson et al.^32^also investigated the efficacy of metronidazole for bacterial vaginosis when given as a vaginal gel (0.75% twice daily for 5 days) and as oral therapy (500 mg twice daily for 7 days). The efficacy of these two formulations was reported to be similar; however, oral therapy was associated with more gastrointestinal complaints. In another study, Levine & Watson^33^ described the pharmacokinetics studies of progesterone gel given via vaginally and oral progesterone. From findings it was suggested that progesterone gel caused greater bioavailability with less relative variability than oral progesterone. Our findings were also in agreement to the findings from previous literature. As hypothesized, vaginal administration of ^99m^Tc-P60+CRB-NBG exhibited higher systemic absorption as compared to oral administration. This could be due to bypass of GI Tract and first pass metabolism, as P60 and CRB are reported to undergo high first pass metabolism.^34^

The pharmacokinetic parameters calculated were also in agreement with the gamma scintigrams. It can be concluded that NBG for P60+CRB showed enhanced antibacterial activity and owing to the nano-droplet size, the formulation could be transported trans-vaginally from vaginal cavity to the systemic circulation.

## Conclusion

Aim of the present work was to prepare NBG encapsulated with P60 and CRB for enhanced antibacterial activity. Optimized oil-in-water NE of P60+CRB was developed that showed a MDS of 58 nm, PDI of 0.2 and zeta potential of -16 mV. To enhance the residence time of the formulation at the site of action, chitosan based gel (1.5%) formulation was developed and characterized for P60+CRB NBG. NBG showed enhanced antibacterial activity (as compared to its aq. and NE counterparts) against *E. coli* as determined via growth curve. *Ex vivo* release studies of P60+CRB NBG performed on porcine vaginal mucosa showed 99% release of P60 after 8 hr while 90% CRB was released after 6 h in simulated vaginal fluid. Preliminary *in vivo* gamma scintigraphy and biodistribution studies were performed by radiolabeling of P60+CRB. Scintigrams indicated the higher uptake of gel from vaginal cavity into the systemic circulation as compared to the aqueous form of P60+CRB which was retained primarily in the vaginal cavity. The pharmacokinetic parameters calculated were also in agreement with the gamma scintigrams. It can be concluded that NBG for P60+CRB showed enhanced antibacterial activity and owing to the nano-droplet size, the formulation could be transported trans-vaginally from vaginal cavity to the systemic circulation.

## Acknowledgments

The authors would like to thank the Department of Biotechnology, Government of India for providing financial support to conduct the research work *(DBT project No.BT/PR7215/NNT/28/654/2013).* The authors are grateful to the Jaypee Institute of Information Technology, Noida, UP (India), for the infrastructural support.

## Ethical Issues

Approval to carry out animal studies was obtained from the INMAS Institutional Animal Ethics Committee (IAEC), New Delhi, India, IAEC vide number INM/IAEC/2012/05 and their guidelines were followed throughout the study.

## Conflict of Interest

All the authors declared that they have no conflict of interest.
